# The development and feasibility of treadmill-induced fall recovery training applied to individuals with chronic stroke

**DOI:** 10.1186/s12883-019-1320-8

**Published:** 2019-05-25

**Authors:** Jamie Pigman, Darcy S. Reisman, Ryan T. Pohlig, Tamara R. Wright, Jeremy R. Crenshaw

**Affiliations:** 1 Department of Kinesiology and Applied Physiology, University of Delaware, Newark, DE USA; 2 Department of Physical Therapy, University of Delaware, Newark, DE USA; 3 Biostatistics Core Facility, University of Delaware, Newark, DE USA

**Keywords:** Balance, Stability, Falls, Rehabilitation, Perturbation training

## Abstract

**Background:**

Exercise has failed to reduce falls in those with chronic stroke. A limitation of traditional exercise is that the motor responses needed to prevent a fall are not elicited (i.e. they lack *processing specificity*). Balance reactions often require compensatory steps. Therefore, interventions that target such steps have the potential to reduce falls. Computerized treadmills can deliver precise, repeatable, and challenging perturbations as part of a training protocol. The objective of this study was to develop and determine the feasibility of such training applied to those with chronic stroke. We developed the training to address specificity, appropriate duration and repetition, and progressive overloading and individualization. We hypothesized that our intervention would be acceptable, practical, safe, and demonstrate initial signs of efficacy.

**Methods:**

In this single-arm study, thirteen individuals with chronic stroke (29–77 years old, 2–15 years post stroke) performed up to six training sessions using a computer-controlled treadmill. Each session had separate progressions focused on initial steps with the non-paretic or paretic limbs in response to anterior or posterior falls. Perturbation magnitudes were altered based on performance and tolerance. *Acceptability* was determined by adherence, or the number of sessions completed. *Practicality* was documented by the equipment, space, time, and personnel. Adverse events were documented to reflect *safety*. In order to determine the *potential-efficacy* of this training, we compared the proportion of successful recoveries and the highest perturbation magnitude achieved on the first and last sessions.

**Results:**

The training was *acceptable*, as evident by 12/13 participants completing all 6 sessions. The protocol was *practical*, requiring one administrator, the treadmill, and a harness. The protocol was *safe*, as evident by no serious or unanticipated adverse events. The protocol demonstrated *promising signs of efficacy*. From the first to last sessions, participants had a higher proportion of successful recoveries and progressed to larger disturbances.

**Conclusions:**

Using a computerized treadmill, we developed an approach to fall-recovery training in individuals with chronic stroke that was specific, considered duration and repetition, and incorporated progressive overloading and individualization. We demonstrated that this training was acceptable, practical, safe, and potentially beneficial for high-functioning individuals with chronic stroke.

**Trial registration:**

Retrospectively registered at clinicaltrials.gov (NCT03638089) August 20, 2018.

## Background

Up to 75% of those living with stroke fall each year [1–3], and individuals with chronic stroke have a fall risk that is twice that of age- and sex-matched peers [4]. Despite the beneficial effect that exercise has had on reducing falls in other populations, such as that of community-dwelling older adults [5], exercise has not reduced falls in those with chronic stroke [6, 7]. Previously considered exercises have focused on standing and walking balance, strength, flexibility, and endurance. A limitation of these exercise interventions is the lack of *processing specificity* [8]*.* In other words, the motor responses needed to prevent a fall are not elicited or effectively modified using traditional exercise methods.

Trips and slips cause one-third of post-stroke falls [9]. Successful recovery from such common perturbations is often dependent upon the skill of compensatory stepping [10, 11]. Individuals with chronic stroke have an impaired ability to recover from anterior [12–15] and posterior [16–18] falls. For example, those with stroke demonstrate lower anterior and posterior multiple-stepping thresholds, defined as the disturbance magnitude that elicits more than one step, compared to peers with no previous stroke [19]. These lower thresholds are associated with a delayed and reduced muscle response of the paretic limb. When a step is taken to arrest a fall, those with chronic stroke generally prefer to step with their non-paretic limb [12, 20, 21], and demonstrate a hopping strategy to avoid bearing weight with the paretic limb [12]. Their recovery steps are characterized by shorter lengths and more trunk rotation, resulting in a less stable foot placement that is closer to the whole-body center of mass [17, 22]. The inability to initiate a recovery step with the paretic limb has been prospectively related to falls in the free-living environment [15]. Therefore, interventions that specifically target fall-recovery steps, including those with the paretic limb, have the potential to reduce falls in individuals with chronic stroke.

The balance reactions of those with stroke are modifiable with practice. Over the course of 15 training sessions, individuals with stroke were able to recover from larger platform surface perturbations with a feet-in-place response [23, 24], also improving weight-bearing symmetry [23]. The extent to which the *stepping response* of those with chronic stroke can be improved with such repetitive practice is not well understood. From the first to second exposure to a simulated slip, those with chronic stroke were able to modify the stepping response of their paretic limb [25]. Kinematic improvements included a more stable position of the whole-body CoM at toe-off of the recovery step, as well as a longer recovery step. This short-term adaptation is an encouraging sign that sustained, and perhaps more profound adaptation could be possible. In a recent report, a six-week perturbation-based intervention for those with chronic stroke resulted in higher scores on the reactive balance subset of the mini-BESTest compared to a control group receiving traditional therapy [26]. This evidence suggests that such training can improve the stepping response. However, the benefit of perturbation-based training on subsequent falls was inconclusive, with no between-group differences in post-training fall rates.

Given that perturbation-based balance training has reduced falls in older adults, individuals with Parkinson’s disease, and those with sub-acute stroke [27, 28], the benefits of such training on those with chronic stroke warrant investigation beyond a single study. In the aforementioned randomized controlled trial [26], balance training consisted of self-initiated tasks and therapist-delivered pulls and pushes, a feasible approach in many settings. Along with its magnitude, the method of applying a perturbation (e.g. surface translations, waist pulls, lean releases) alters the response to the perturbation and influences the degree to which responses reflect balance impairment [29]. It is reasonable to explore whether other methods of delivering perturbations, especially those that allow for large-magnitude disturbances, could elicit greater benefits in terms of reducing the risk of falls. Computer-controlled treadmills have been used to implement controlled, repeatable, and challenging perturbations [30–34]. Such treadmill-induced falls necessitate stepping responses similar to that of overground trip and slip recovery [35, 36]. To our knowledge, however, this form of fall-recovery training has not been developed for use with the chronic stroke population. Given demands of this approach that differ from that using therapist-induced perturbations, we do not know if computer-controlled treadmill training would be feasible in this population.

The objective of this study was to develop and determine the feasibility of fall-recovery training applied to those with chronic stroke using a computerized treadmill. We developed the training to address specificity, appropriate duration and repetition, and incorporate progressive overloading and individualization. Given the feasibility and effectiveness of such training with other populations, as well as the apparent feasibility of other forms of perturbation-based training with those with chronic stroke [26], we hypothesized that our intervention would be acceptable, practical, safe and demonstrate initial signs of efficacy [37].

## Methods

### Participants

This study recruited from the University of Delaware’s Stroke Studies Registry, which houses over 850 participants, with continual recruitment efforts through support groups, therapy clinics, and advertisements. Basic clinical measures of balance and falls self-efficacy are recorded upon registry enrollment, allowing us to target potential participants. We attempted to contact 30 individuals that appeared to meet study inclusion and exclusion criteria. Inclusion criteria included an age of 18 years or older, having had a single stroke of non-cerebellar origin, and a self-reported ability to walk a city block without a gait aid such as a walker or cane. Exclusion criteria included other neurologic disorders, musculoskeletal surgeries within the past year, recent cardiovascular events, or other conditions that preclude safe participation. Of those contacted, thirteen individuals (10 males, 3 females) (Table 1) with chronic stroke participated in this study. Those who were 50 years of age or older underwent a Dual-energy X-ray absorptiometry (DXA) screening to ensure that they were not osteoporotic (total hip or femoral neck bone mineral density t-score < − 2.5) [38]. This screening criterion, which has been used previously in studies of older adults [39], was conservatively in place to reduce the risk of fractures from the impact of fall-recovery steps or falls into the safety harness. No individuals were excluded from the study due to DXA screening. Seven of the 13 participants reported at least one fall within the year prior to study enrollment. All participants provided written informed consent to participate in this study.

**Table 1:** Demographic and clinical assessment data (*n* = 13)

Measure	Mean (SD), Range
Age (Years)	57 (13), 29–77
BMI (kg/m^2^)	28.3 (3.6), 22.0–33.0
Years after stroke	5 (4), 2–15
Fugl-Meyer LE	24 (6), 8–32
Activities Specific Balance Confidence Scale (ABC)	91 (8), 76–100
Functional Gait Assessment (FGA)	17 (5), 9–29
Berg Balance Scale (BBS)	51 (5), 38–56

### Fall-recovery training principles and development

Our training integrated established principles of exercise prescription [40–42] and neuroplasticity [43] by emphasizing training 1) *specificity*, 2) *duration* and *repetition*, and 3) *progressive overloading* and *individualization*.

#### Specificity

Training programs must have specific targets that directly cause or contribute to accomplishing a desired goal [40–42]. Targets of an exercise program may include specific muscle actions, speed of movements, or movement patterns [42]. The perturbations delivered within our training were designed to target these aspects, necessitating the rapid, coordinated stepping response similar to that of trip- and slip-recovery. In response to a trip, the stance limb plantarflexors activate to lengthen recovery steps and reduce forward angular body momentum and trunk rotation [12, 35, 44–46]. Rapid treadmill belt accelerations, directed posteriorly, require a similar recovery response to that of trip-recovery [35, 46]. In addition, large displacements that can be delivered using a treadmill require multiple steps to recover balance, a response that aligns with the multistep response of trip-recovery [47]. In response to a slip, the lower extremity muscles must execute a coordinated response (i.e. one not characterized by co-contraction) [48], placing the recovery step posterior to, but not too far laterally from the whole body center of mass [11, 49–51]. This type of fall can be simulated using rapid, anteriorly-directed surface translations [36, 52]. Given the similarities between overground and treadmill-induced fall recoveries, using the latter method can be an integral and valid exercise approach to reducing falls in those with chronic stroke.

In accordance with the principle of specificity, focusing on anterior *and* posterior falls may be necessary to specifically reduce both trip- and slip-related falls. Older women who underwent training focused on anterior fall-recovery reduced the rate of trip-related falling in the laboratory by 86% [53]. This form of training also reduced trip-related falls in the free-living environment (rate ratio 0.54, 95% CI 0.30–0.97) [54]. Of note, not all fall causes were reduced with training, suggesting that the benefit was specific to the trip-recovery response. Training with community-dwelling older adults that focused on the posterior fall recovery using a computerized treadmill was effective at improving overground slip-recovery compared to a control group that did not perform training [55]. We do not know, however, if there are specific or general benefits of slip-recovery training to falls in the free-living environment.

When a step is taken to arrest a fall, those with chronic stroke generally prefer to step with their non-paretic limb [12, 20, 21]. The inability to take a recovery step with the paretic limb has been prospectively related to falls in the free-living environment [15]. Trips and slips occurring outside the laboratory may require initial steps with either limb, depending on which limb was perturbed. In other asymmetrically impaired populations, such as those with lower extremity amputations, the kinematic benefits of fall-recovery training are dependent upon the initial stepping limb [33]. In addition, the interlimb transfer of benefits from perturbation-based training may be limited, even in unimpaired participants [56]. Given this evidence, fall-recovery training must include stepping responses from each limb.

The previous application of perturbation-based fall-recovery training in those with chronic stroke [26] followed many of these specificity guidelines. Using therapist-induced perturbations, they induced falls that required anterior, posterior, and lateral stepping. However, single-step responses were encouraged, which may limit any training benefit to the second step. They also blocked preferred stepping limbs in order to encourage stepping with the contralateral limb.

#### Training duration and repetitions

In order to induce plasticity there needs to be a sufficient number of practiced repetitions [43]. The total number of perturbations applied in previous studies have varied greatly, ranging from fewer than 100 perturbations to more than 1000 [26, 27, 31–33, 57]. A previous study of individuals with sub-acute stroke administered a total of six training sessions lasting 30–60 min in duration, with participants tolerating up to 30 lean-release perturbations per session [58]. In the previous application of training to those with chronic stroke, participants attended a mean of 10.5 sessions, with a mean of 55 perturbations per session [26]. We assume that the benefits of training are improved with more sessions, more perturbations, and longer session durations. However, increasing these aspects reduces the likelihood of tolerance. Guidelines in terms of the number and duration of fall-recovery training sessions remain unclear.

#### Progressive overload and individualization

In order to maximize training adaptations, the stimulus must become progressively more challenging [40–42]. Conversely, the training intensity must be tolerable and appropriate to each individual’s abilities [42]. We have previously implemented a progressively challenging training protocol for individuals with lower extremity amputations [33]. Here, the disturbance magnitudes (i.e. initial acceleration) were increased or decreased on subsequent trials depending on whether or not the participant engaged a safety harness. In the previous application of training for those with chronic stroke, therapists delivered pulls and pushes so that “failed” responses of grabbing an object, assistance from the therapist or harness, or multiple steps occurred for ≈50% of trials. This “failure rate” ensured a challenging, participant-specific approach. If tolerance must be prioritized, the magnitude of the perturbation may be limited without substantially affecting efficacy. Training with smaller disturbances can yield benefits not different than that of larger disturbances [59, 60].

### Fall-recovery training implementation

**Fig. 1:**
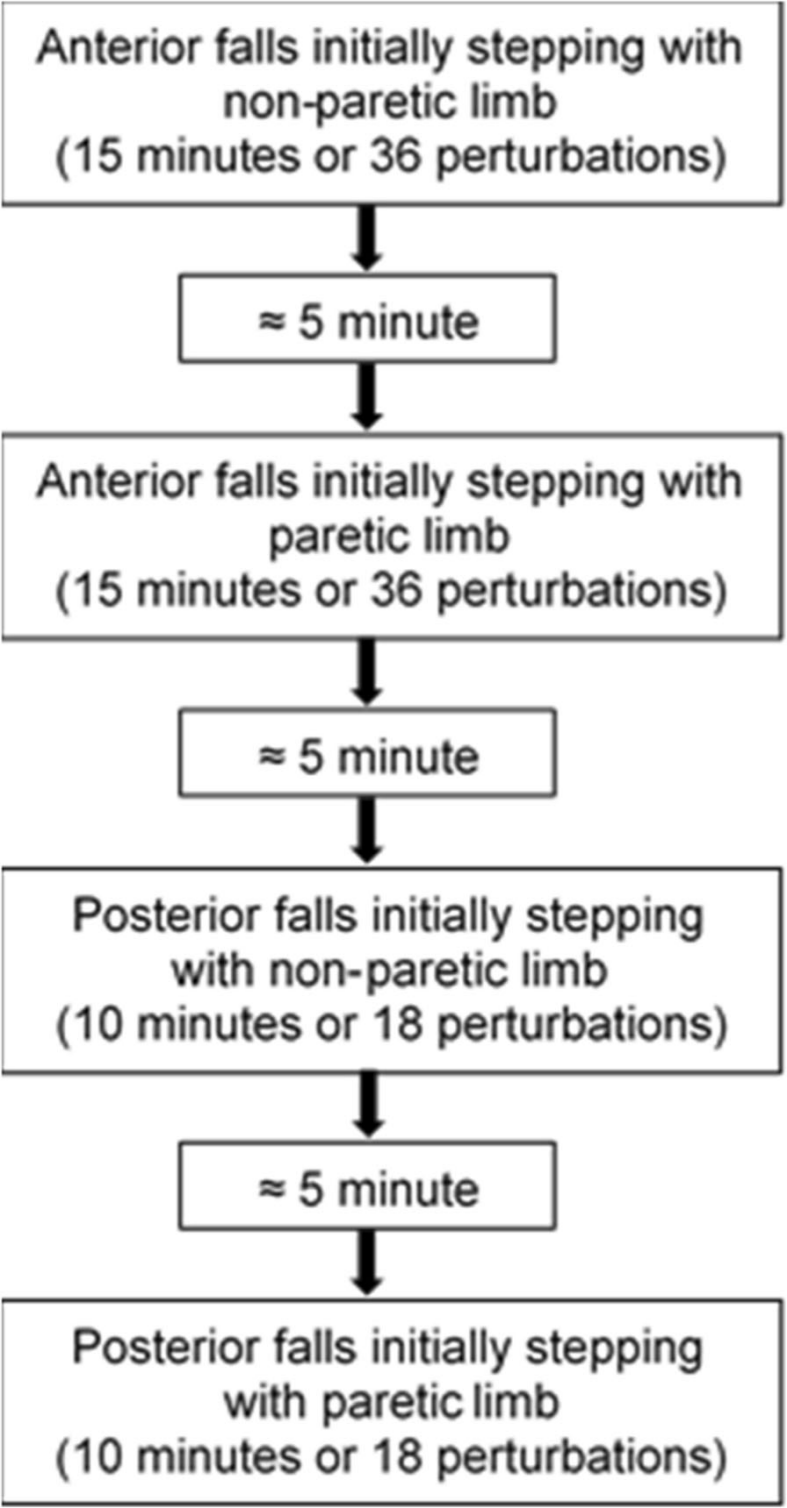
A flowchart depicting a single fall-recovery training session. Participants performed training in this order for each of the six training sessions

This was a single-arm study in which all participants received training. Our training sessions presented here consisted of four progressions of treadmill belt displacements that induced an anterior or posterior fall. Simulated trips and slips were delivered using a commercially available computer-controlled treadmill (ActiveStep®, Simbex, Lebanon, NH). The four progressions within a training session were: 1) anterior falls while stepping with the non-paretic limb, 2) anterior falls while stepping with the paretic limb, 3) posterior falls while stepping with the non-paretic limb, and 4) posterior falls while stepping with the paretic limb (Fig. 1). These progressions were repeated across six sessions over 3 weeks, with participants training two non-consecutive days per week. Each progression that focused on trip-related falls was limited to either 15 min or 36 perturbations, whichever occurred first. Similarly, slip-recovery training was limited to 10 min or up to 18 perturbations per progression. Rest periods lasting approximately 5 minutes occurred in between each progression or at the participant’s request. These progression durations were determined in order to reasonably limit fatigue and to keep training sessions to approximately 1 hour.

Participants were instructed to wear comfortable clothing for exercise, such as a pair of shorts and a t-shirt. They were also instructed to wear well-cushioned, closed-toe athletic shoes with no elevated heels. When awaiting a disturbance, participants stood with their feet placed at a comfortable width, toes evenly positioned in the anteroposterior direction. They were outfitted with a full-body safety harness (Delta™, Capital Safety, Bloomington, MN) attached to a custom-built overhead rail system. Of note, this treadmill system is also available with its own harness system. The support straps were adjusted so that the participant’s hands and knees could not come into contact with the treadmill if there were to be a fall. The harness was instrumented with a force transducer (Dillon, Fairmont, MN), the peak forces of which were recorded for each trial by the study team member. No handrails were available for use on the treadmill.

For trip-training progressions, the disturbance velocity waveforms consisted of an initial, 500 ms acceleration followed by a deceleration phase at − 0.38 m/s^2^. Participants were instructed to “try not to fall” in response to these forward falls, specifically stepping with a targeted limb. The first disturbance of each progression had an initial acceleration of 0.5 m/s^2^, resulting in a displacement of 0.06 m. After a successful recovery, the subsequent disturbance had an initial acceleration + 0.25 m/s^2^ greater than the previous disturbance [33]. After a failed recovery, the subsequent trial acceleration was reduced by 0.25 m/s^2^. Failures were defined as recoveries in which the force transducer recorded more than 20% body weight [61] or recoveries in which the participant stepped with the wrong limb. These treadmill displacements were 15 m or less, accompanied by peak velocities of 3.25 m/s or less and initial accelerations of 6.5 m/s^2^ or less. Each disturbance was preceded by a 1–5 s delay in order to limit pre-planned timing of the response. Additionally, small disturbances (0.3 ms duration, 0.03 m displacement) resulting in a posterior fall were introduced approximately once every six trials to limit anticipatory adjustments.

For the slip-training progressions, the disturbance velocity waveforms were triangular in shape, consisting of 200 ms acceleration and deceleration phases. Participants were instructed to “try to recover in one step” in response to these posterior falls. This directional discrepancy in step constraints was due to previous research suggesting that slip recovery is primarily dictated by the first-step [51], unlike the multiple-stepping response of common trip recovery strategies [47]. The first disturbance of each progression had an initial acceleration of 0.5 m/s^2^, resulting in a displacement of 0.01 m. As with trip-recovery training, the subsequent trial was altered based on recovery success, with between-trial increments of 0.5 m/s^2^. Failure criteria was consistent with trip-recovery training, with the additional case of two or more steps considered as failure. Treadmill displacements simulating a slip were 0.64 m or less, peak velocities were 3.2 m/s or less, and peak accelerations were 16 m/s^2^ or less. As with the trip-recovery training progressions, each disturbance was preceded by a 1–5 s delay, and small disturbances (0.5 ms duration, 0.05 m displacement) resulting in an anterior fall were introduced approximately once every six trials to limit anticipatory adjustments.

Participants were asked to inform research staff if the training intensity became too much for them to tolerate (i.e. muscle soreness, general fatigue, or uneasiness being on the treadmill). In such cases, training continued at the highest disturbance magnitude tolerated for the remainder of the session. This approach was intended to limit nervousness or discomfort and maintain compliance while promoting practice repetitions. In subsequent sessions, we would attempt to increase the disturbance magnitude tolerated.

All participants attempted to complete six sessions of the training protocol as described in the previous section. Of note, one participant wore an articulating ankle foot orthosis (AFO) during training that was typically worn on a day-to-day basis for ankle stability and safety.

## Outcomes

### Feasibility & Limited Efficacy

In order to determine the feasibility of our protocol, we evaluated the *acceptability*, *practicality*, and *safety* of our proposed intervention [37]. *Acceptability* was defined as how the individuals reacted to the intervention [37]. We evaluated this by examining 1) adherence to the training, which was defined as the number of training sessions completed out of the six training sessions; and 2) number of trips and slips performed within sessions for each limb. For sessions or progressions that were cut short, we detailed subject-reported or other reasons. *Practicality* is the extent to which an intervention can be delivered when resources, time, and/or participant commitment are constrained in some way [37]. In our study *practicality* was documented by the equipment, space, time (participant and personnel), and number of personnel needed. *Safety* was determined by tracking adverse events. The definition of a serious adverse event was any undesirable experience associated with the training that resulted in death, hospitalization, disability, or permanent damage, or required intervention to prevent permanent impairment or death. A non-serious adverse event was any incident that caused a participant to temporarily stop or halt training [62]. To be clear, we anticipated non-serious adverse events of muscle soreness and nervousness based on our previous experience administering treadmill-delivered perturbations to those with lower extremity amputations [33] and older adult women [39].

*Limited-efficacy testing* for interventions is often conducted with a convenience sample, intermediate rather than final outcomes, shorter follow-up periods, and/or with limited statistical power [37]. This research used a convenience sample from the target population to perform the training protocol as outlined above. In order to determine the potential efficacy of this training we compared the proportion of successful recoveries on the first and the last sessions. We also compared the highest perturbation magnitude from which a successful recovery was achieved from the first and the last sessions. Researchers were not blinded to these outcomes. Cases where the disturbance magnitude was limited as a safety precaution were removed from the analysis. Cases where the disturbance magnitude was limited due to anxiety were still considered, however, as this psychosocial influence is relevant to the risk of falls [63] and observed physical activity outside of the laboratory [64, 65].

## Results

### Acceptability and safety

Twelve out of the thirteen participants successfully completed all six training sessions. There were no serious adverse events. One participant only completed five sessions due to an acute illness prior to the sixth session, and scheduling conflicts prevented the rescheduling of the sixth session in a timely manner (greater than 30 days). There was one non-serious anticipated event involving a participant needing to delay training due to minor muscle soreness of their non-paretic hip after the second training session. After approximately 3 days of rest, the participant reported that the soreness had subsided. They resumed training and successfully completed all six sessions without further reports of hip soreness.

Across the entire study, participants demonstrated the ability to perform a similar number of simulated trip repetitions with their non-paretic (mean (SD) = 20.0 (5.8), range: 10–33), and paretic limbs (mean (SD) = 20.3 (6.5), range: 9–36). Additionally, the number of repetitions in slip-recovery training were similar for non-paretic-limb steps (mean (SD) = 13.7 (2.0), range: 9–17) and paretic-limb steps (mean (SD) = 13.1 (1.9), range: 8–16).

On a subject-by-subject basis, the magnitude of the disturbance was limited in order to prevent injury or maintain adherence. For example, when we observed one participant demonstrating slight ankle inversion during trip-recovery steps, we limited the progression to an initial acceleration of 4.25 m/s^2^ as a conservative safeguard against ankle injury. Another participant self-reported nervousness about being on the treadmill, so we limited the training progression until the participant was comfortable doing so. For this participant, trip and slip training was reduced to 5-min for each limb. The duration was gradually increased to 10-min for each limb from the second to the sixth session.

Five participants experienced controlled laboratory falls, fully engaging the safety harness in response to simulated trips. Three of these participants only experienced one fall, while the other two participants experienced multiple falls across training. The initial treadmill belt acceleration associated with these falls into the safety harness ranged from 1.5 m/s^2^ to 4.5 m/s^2^. One participant experienced three falls into the safety harness in response to simulated slips. The initial treadmill belt acceleration of the slip-induced falls ranged from 4.5 m/s^2^ to 5.0 m/s^2^.

### Practicality

At the beginning of this study, we assigned two research team members for each training session. One person operated the computer-controlled treadmill delivering the perturbations. This computer was located approximated two meters away from the treadmill. The second person was positioned near the treadmill and interacted with the participant directly, making sure, the participant was positioned in the center of the treadmill prior to each perturbation, confirming that the participant was comfortable, and answering any questions from the participant. With experience, we determined that we could omit this second team member and conduct training sessions with one team member operating the computer and interacting with the participant. This individual was trained to operate all equipment, and they were CPR and first-aid certified. In this case, the team member was a graduate student in the University of Delaware Biomechanics and Movement Sciences program. Inclusion and exclusion criteria, however, were confirmed by a licensed physical therapist and a certified DXA operator, as per Delaware regulations, if needed.

### Limited-efficacy testing

**Fig. 2:**
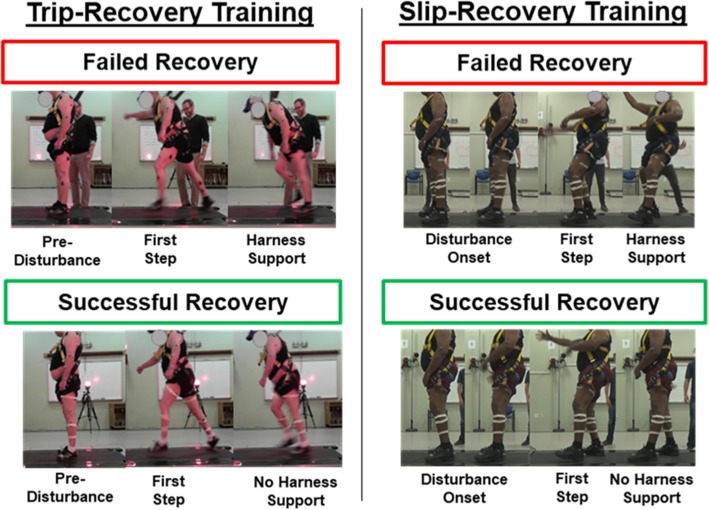
Individuals with chronic stroke participate in trip-recovery (left) and slip-recovery (right) training. Treadmill-induced disturbances were applied to standing participants, necessitating steps to prevent a fall into a safety harness. On the left, a participant fell in response to a simulated trip (*a* = 4.5 m/s^2^) on the first training session. On the last training session, he successfully recovered from the same disturbance, initially stepping with his paretic limb. On the right, a participant fell in response to a simulated slip (*a* = 5.0 m/s^2^) during the first session. On the last training session, he successfully recovered from the same disturbance, initially stepping with his non-paretic limb

Our limited preliminary data suggest that fall-recovery performance may be improved in those with chronic stroke*.* From the first to last sessions, thirteen participants successfully recovered from a higher proportion of falls and progressed to larger disturbance magnitudes (Table 2). By the end of training, participants also successfully recovered from the same disturbance magnitude that originally caused them to fall (Fig. 2).

**Table 2:** Training-based changes in the proportion and magnitude of successful fall recoveries

Fall Direction	Initial Stepping Limb	Variable	First Session	Last Session	Change w/ training	Cohen’s d
Anterior (Simulated Trips)	Non-Paretic	% Successful Trials (%)	91 (14)	99 (3)	8 (11)	0.73
		Largest Disturbance (m/s^2^)	3.6 (0.9)	4.0 (1.0)	0.4 (0.9)	0.44
	Paretic	% Successful Trials (%)	77 (29)	89 (27)	13 (16)	0.81
		Largest Disturbance (m/s^2^)	2.9 (1.4)	3.4 (1.4)	0.4 (0.4)	1.00
Posterior (Simulated Slips)	Non-Paretic	% Successful Trials (%)	79 (14)	90 (10)	11 (11)	1.00
		Largest Disturbance (m/s^2^)	3.9 (1.2)	4.5 (1.2)	0.6 (0.6)	1.00
	Paretic	% Successful Trials (%)	60 (26)	75 (16)	15 (18)	0.83
		Largest Disturbance (m/s^2^)	2.9 (1.3)	3.2 (1.1)	0.3 (0.5)	0.60

Note: First session, last session, and change with training data are displayed as mean (SD)

## Discussion

The objective of this study was to develop and determine the feasibility of fall-recovery training using a computer-controlled treadmill applied to those with chronic stroke. We hypothesized that our intervention would be acceptable, practical, safe and demonstrate initial signs of efficacy [37]. We demonstrated that our intervention was acceptable for most participants, practical given our resources, and capable of eliciting positive effects at a limited scale.

We determined that this intervention was acceptable and safe for nearly all of our participants. None of the thirteen participants enrolled in this training dropped out due to training-related causes. Some, but not all previous perturbation-based intervention studies have reported adherence results. In a study of trip-recovery training of older adult women, 82 of 84 participants completed the training, although the reasons for participant dropout were not reported [54]. In an agility-based exercise intervention of those with chronic stroke, 11 of 59 participants discontinued the intervention due to time commitments, injury, illness, or unspecified personal reasons [66]. The previous application of perturbation-based training to those with chronic stroke had incidents of fatigue, joint pain, and delayed onset muscle soreness, although these outcomes were also apparent in a control group receiving traditional exercise [26, 28]. A recent study evaluating the perturbation response of older women in a single session resulted in 10 of 112 participants ending participation due to nervousness or soreness [39]. From these results, we anticipate that a small (< 15%) rate of dropout may occur in our intervention when applied at a larger scale, a rate that may increase if applied to a lower-functioning cohort. For those who do drop out, we suggest that the intervention focus on the underlying factors that limited participation, such as the fear of falling which underlies anxiety or the orthopedic issues, which underlie soreness. Of note, we encourage future studies to document acceptability and the reasons for dropout, as these factors represent a critical barrier to administering new interventions in a clinical setting.

An aspect that helped to maintain adherence was our participant-specific approach, altering training difficulty based on the performance *and* tolerance of each participant. The single participant who demonstrated nervousness, likely associated with a fear of falling, was able to increase the session duration over the course of training. Exercise has been shown to be an effective treatment for fear of falling [67], yet the effects of perturbation-based fall-recovery training on psychosocial factors are not well known. Given our task-specific approach, we hypothesize that there likely are benefits to balance confidence, falls self-efficacy, and the fear of falling.

Aside from the specialized treadmill, this protocol required little space and few staff to administer it. To our knowledge, this study is the first to demonstrate the feasibility of treadmill-induced fall-recovery training applied to individuals with chronic stroke. Unlike training using therapist-induced perturbation [26, 28] our treadmill perturbations are more precise and repeatable within and across administrators. This level of control allows for the consistent application and objective analysis of performance-based measures within and across training sessions. The cost and availability of our treadmill, however, is a barrier to its broad application. We anticipate, however, that costs will be reduced and availability will be increased with time.

With training, participants recovered from larger disturbances and a greater proportion of disturbances. This result confirms previous reports that the stepping response to a fall can be improved with practice in those with chronic stroke [26]. We do not yet know the biomechanical or neurological adaptations that underlie this improvement in fall-recovery skill. Individuals with a unilateral lower-extremity amputation, another unilaterally impaired population, improved their anterior fall-recovery response with a similar training program [33]. Improvements from simulated-trip training included an increased initial step length and a reduced trunk flexion angle, with benefits limited to steps with the prosthetic limb [33]. We hypothesize that our training elicited similar improvements, with benefits likely specific to stepping with the non-paretic or paretic limb.

Our training did not fully integrate the principle of *variation* in exercise prescription [40–42]. During training, the participants were aware of the disturbance type (i.e. trip or slip) and they understood that the disturbance magnitudes would get progressively more challenging as the training session proceeded. Perhaps, in future iterations of this intervention, later-stage training sessions could integrate anterior and posterior disturbances of various magnitudes to promote variation. In addition, we did not deliver lateral disturbances in our protocol. Post-stroke individuals have an impaired response to falls in all directions [68]. This response is profoundly impaired when the fall is toward the paretic limb [19, 69]. We focused on anterior and posterior falls in an attempt to simulate trips and slips, fall-causes that account for one-third of falls in this population [9]. Although the perturbations were applied in the anteroposterior direction, such disturbances do challenge frontal plane stability. In previous studies, mediolateral step placement was an important aspect of the recovery from anterior and posterior disturbances [51, 70]. We excluded lateral perturbations in order to minimize time and fatigue, but such perturbations can be delivered using our treadmill [71].

Most falls for community-dwelling individuals with stroke occur while walking [3, 4, 72–74]. We applied our perturbations, however, as participants were standing. Practicing fall-recovery during walking may improve the specificity of our approach. Doing so, however, presents challenges related to training goals and hardware limitations. One goal of our approach was to focus on recovery steps with the non-paretic and paretic limbs. Although our treadmill is able to deliver walking disturbances relative to gait events, it is not able to discriminate left and right steps. Therefore, we could not administer a limb-specific, progressively challenging series of disturbances. With between-limb differences in stepping ability, a progression that was not limb-specific would likely be too challenging for steps with the paretic limb or not challenging enough for steps with the non-paretic limb. By delivering surface translations while the participants were in a static standing position, we could isolate and instruct them to step with a specific limb. Given the observed benefits of other studies that delivered standing perturbations [33, 53], we believe that the benefits of delivering perturbations while standing outweigh its limitations.

Many of our participants were high-functioning and active individuals (Table 1). Therefore, we cannot assume that this approach is feasible with lower functioning participants, particularly those with a high fear of falling, low falls self-efficacy, and those that rely on walking aids such as a cane or walker. Given that the effects of stroke are dependent on the injury location and severity, initial fitness of the person, and intensity of previous rehabilitation, this population presents with a wide range of function. Aspects such as lower-extremity impairment or age may alter responsiveness to our training. These factors, then, would serve as ways to stratify groups in a controlled experiment. Further study is needed to identify such factors. Walking aids are commonly used by individuals that have had a stroke [75, 76]. The effectiveness and utility of using a cane to recover from a fall are not fully understood. In some cases, using a cane has been shown to impede compensatory steps needed to successfully recovery from lateral [77] and posterior [78] falls. Those with Parkinson’s disease improved fall-recovery in response to an unpracticed simulated slip using a cane, but the beneficial effects of using the cane were only observed during the initial perturbation exposure [79]. The feasibility, effectiveness, and utility of training those with stroke who rely on walking aids requires further study.

Our study was limited by not being a controlled experiment, so the limited-efficacy results should be interpreted with caution. Our outcomes were variables measured within training sessions. Therefore, the addition of reliable, yet precise pre-training and post-training balance measures would be needed to conduct such a study. Without an active control group, we cannot conclude that benefits would be due to the training itself. It may be that confounding influences, such as interactions with study staff or the general benefits of more activity underlie balance improvements. To clarify, our aim was not to conduct a “feasibility trial”, with the goal of determining whether a future randomized controlled trial could be done, should be done, and, if so, how [80]. Aspects of a feasibility trial not considered here include the willingness of participants to be randomized, the willingness of clinicians to recruit participants, the number of eligible participants, and characteristics of the outcomes measured outside of the intervention. These aspects are specific to the planned randomized controlled trial. Our focus was on the feasibility of the intervention itself. Certainly, this intervention should be studied within a statistically powered, controlled trial. However, our preliminary results can inform the development of such trials, as well as the development of clinical applications of this training.

## Conclusions

In summary, we have determined that fall-recovery training using a computer-controlled treadmill is an acceptable and practical exercise intervention for higher functioning individuals with chronic stroke. Our initial results suggest that the training can be beneficial for this population. We intend to extend this work, evaluating the neural and biomechanical benefits of the training, applying it to a larger cohort with more impaired function, and evaluating its effects on balance self-confidence, walking activity, and subsequent falls in the free-living environment.

## Abbreviations

ABC: Activities of balance confidence scale
AFO: Ankle foot orthosis
BBS: Berg balance scale
BMI: Body mass index
CoM: Center of mass
DXA: Dual-energy X-ray absorptiometry
FGA: Functional gait assessment
Fugl-Meyer LE: Lower extremity
kg: Kilograms
m: Meters
m/s: Meter per second
m/s^2^: Meters per second squared
ms: Milliseconds
s: Seconds
SD: Standard deviation
